# A Ward-Based Mindfulness in Nature Group, as an Intervention to Reduce Stress for Patients and Staff on the Mother and Baby Unit

**DOI:** 10.1192/bjo.2026.11465

**Published:** 2026-06-30

**Authors:** Kate Preston, Karl Scheeres

**Affiliations:** Avon and Wiltshire Mental Health Partnership NHS Trust, Bristol, United Kingdom

## Abstract

**Aims:**

A literature review search ran in April 2025 over five databases (Cinahl, Emcare, Medline, Cochrane Library, PsycInfo) showed there is substantial high-level evidence supporting the use of mindfulness-based interventions (MBIs) for perinatal depression and anxiety.

Nature exposure has been shown to have protective effects on mental health among both general adults and perinatal populations. However, there is insufficient existing research to draw firm conclusions about the effects of nature-based interventions (NBIs) on perinatal mental health. No studies had been found that combine MBIs with NBIs for the perinatal population. A ward-based mindfulness group was devised with the aim to provide a stress-relieving space for patients and staff.

**Methods:**

A Mindfulness in Nature group was developed and led on New Horizon Mother and Baby Unit in Southmead, Bristol from March until July 2025. Ward staff, patients, their babies, and visiting relatives were invited take part. Sessions were led outdoors in the ward garden. Sessions followed a common structure and began with a poetry reading. Participants were then invited to take part in 2–3 group and individual mindfulness activities. Each week the group ended with a guided mindfulness closing practice. Quantitative data was collectedvia pre- and post-session feedback forms which were filled out contemporaneously over 8 weeks. Participants were asked to rate how stressed they felt on a scale of 0–5 before and after the session. They were also asked to rate how they found the session via a face rating scale. Qualitative feedback was collected to gain participants views on the sessions.

**Results:**

Eighteen feedback forms were collected from seven sessions by ten participants. Two forms were partially completed and could not be counted. Two completed forms were by staff. Every participant rated the session as being “good” (smiling face). Eleven forms showed a reduction by 1 in participant stress levels after the session, three forms showed reduction by 2 points, and one form reduction by 3 points. One participant rated their stress as the same before and after the session. None reported an increased level of stress post-session.

**Conclusion:**

Participation in a ward-based mindfulness in nature group reduced self-reported stress levels of staff and patients who attended. Positive qualitative feedback was also received. More rigorous studies would be indicated to assess the efficacy of mixed nature and mindfulness-based interventions for improving symptoms of stress, depression and anxiety for the perinatal patient and staff populations.

